# Evaluation of the Effectiveness of Surgical Treatment of Malignant Glaucoma in Pseudophakic Eyes through Partial PPV with Establishment of Communication between the Anterior Chamber and the Vitreous Cavity

**DOI:** 10.1155/2015/873124

**Published:** 2015-08-12

**Authors:** Marek Rękas, Karolina Krix-Jachym, Tomasz Żarnowski

**Affiliations:** ^1^Ophthalmology Department, Military Institute of Medicine, Szaserów Street 128, 04-141 Warsaw, Poland; ^2^Department of Diagnostics and Microsurgery of Glaucoma, Medical University of Lublin, Chmielna Street 1, 20-079 Lublin, Poland

## Abstract

*Purpose*. Determination of partial posterior vitrectomy (PPV) in the proposed modification for treatment of malignant glaucoma. *Methods*. The prospective, consecutive, single-center case series study involved patients in whom symptoms of malignant glaucoma occurred after combined cataract and glaucoma surgery or after glaucoma surgery in pseudophakic eye. When medical and laser treatment were not successful, partial PPV with establishment of communication between the anterior chamber and the vitreous cavity was performed. Efficacy measures were intraocular pressure (IOP) reduction, corrected distance visual acuity (CDVA), AND the number of antiglaucoma medications. Surgical success and occurring complications were also evaluated. *Results*. The study enrolled 20 eyes of 17 patients. Average IOP was reduced from 30.4 ± 14.2 (SD) mmHg to 14.6 ± 3.2 (SD) mmHg one year after surgery (*P* < 0.00001). A statistically significant reduction of the number of antiglaucoma medications was obtained from 3.3 ± 1.1 (SD) preoperatively to 1.2 ± 1.1 (SD) at the end of follow-up. Statistically significant improvement of CDVA was observed 3, 6, and 12 months after surgery. *Conclusions*. Partial PPV with establishment of communication between the anterior chamber and the vitreous cavity enables effective IOP control over a 12-month observation; however, in most cases, it is necessary to use antiglaucoma medications for IOP control.

## 1. Introduction

Surgical treatment of malignant glaucoma is based on interrupting the sequence of events lying at the foundation of its pathophysiological process. In the classical form of this complication, aqueous humor accumulates in the area of the vitreous cavity due to ciliary block, and, as a result of this, there is an increase in the vitreous pressure that is transferred to the structures of the anterior segment causing a forward movement of the lens-iris diaphragm [1]. Most often, malignant glaucoma develops quickly, within days after primary surgery with a clear cause-effect relationship with the performed procedure, although its occurrence can also be postponed [2–4]. Recurrences of malignant glaucoma are observed both after PPV that was initially effective and after conservative treatment [5–8]. It seems that the characteristic clinical picture of malignant glaucoma with recurrences is caused by the preservation of the primary mechanism that is found at the basis of aqueous humor accumulation in the posterior segment of the eye.

Observations resulting from the application of PPV for the treatment of malignant glaucoma in pseudophakic eyes make it possible to state that elimination of adhesion of the vitreous body, lens capsule, and its zonula to the plane of the iris, pupil, and iridectomy restores normal aqueous humor flow from the posterior to the anterior segment of the eye [6, 9]. While PPV unburdens the vitreous chamber and improves anatomical proportions in the anterior chamber, preservation of communication between the posterior and anterior segments of the eye is decisive to the stability of this procedure.

Surgical treatment in malignant glaucoma is usually applied when conservative and laser treatment do not have the intended effect [6, 9, 10]. On the other hand, the time that passes from diagnosis has an impact on the nature of complications and the effectiveness of surgical procedure. Certain authors stress that the greatest chance for permanent success of PPV in the case of malignant glaucoma is related to quick implementation of surgical treatment [11].

The goal of the study was to evaluate surgical treatment of malignant glaucoma in pseudophakic eyes by partial PPV extended by communication of the anterior chamber and vitreous cavity through removal of zonules and of the anterior and posterior capsule in the area of the iridectomy which was formed during primary glaucoma surgery.

## 2. Materials and Methods

### 2.1. Patients

The following treatment schedule was applied when symptoms of malignant glaucoma occurred: mannitol 2 g per kg intravenously once or twice a day, acetazolamide 250 mg* tid*, and locally: atropine 1%* qid*, Cosopt (dorzolamide hydrochloride-timolol maleate ophthalmic solution)* bid*, and 0.1% dexamethasone phosphate* tid*. The next step was laser treatment (capsulotomy through the iridectomy). These methods were of temporary effect in most cases. Sustainable improvement after medical and laser treatment was obtained in only 2 cases (9.1%). Regardless of using all available methods most of malignant glaucoma cases (20 eyes) needed to be treated surgically because of a weak effect of conservative treatment. The indication for surgical treatment was diagnosis of malignant glaucoma in eyes after cataract and glaucoma surgery or after glaucoma surgery in pseudophakic eyes based on symptoms such as progressive increase of IOP together with axial shallowing of the anterior chamber in the presence of a patent iridotomy, despite the application of conservative and laser treatment [6, 9, 10, 12, 13].

All patients required IOP reduction and restoration of anatomical relationships to prevent further progression of malignant glaucoma and its complications.

Exclusion criteria included suprachoroidal hemorrhage, pupillary block, hypotony progressing with shallowing of the anterior chamber or choroidal detachment, and inflammation in the anterior or posterior segment of the eyeball requiring conservative or surgical procedures of a different type.

Seventeen patients (20 eyes) with symptoms of malignant glaucoma occurring at various time after primary surgery were recruited into the study.

### 2.2. Preoperative Examination

During qualification, medical history concerning prior procedures and applied treatments was taken. During baseline examination, IOP, uncorrected distance visual acuity (UDVA), and corrected distance visual acuity (CDVA) were measured, the anterior segment was examined with consideration of anterior chamber depth, eye fundus examination along with evaluation of c/d and measurement of central corneal thickness and axial length (AXL) was performed, and gonioscopic examination was conducted. The anterior and posterior segments were evaluated in terms of fulfillment of the inclusion criteria.

### 2.3. Surgical Technique

All surgeries were performed with retrobulbar anesthesia (2% xylocaine and 0.5% bupivacaine) by one surgeon (M.R.). The anterior chamber was opened (1.2 mm) at the 5:00 or 7:00 position depending on the eye that was being operated on. The anterior chamber was filled with viscoelastic (Viscoat, Alcon Laboratories, Inc.). A trocar was inserted into the vitreous cavity through the pars plana at a distance of 3.5 mm from the corneal limbus, and after that 25-gauge PPV was performed, which included removal of the anterior part of the vitreous body. After the vitreous chamber was unburdened, the anterior chamber was filled using Viscoat, adhesions around the present iridectomy, iris-lens adhesions, iridocorneal adhesions, and iris-capsule adhesions were released, and then the anterior chamber was opened in the projection of the iridectomy. A 25-gauge needle was inserted through this opening, puncturing the anterior and posterior capsule in the area of the iridectomy. There are few possible ways (through the anterior chamber or from behind) to clear the iridectomy and all of these methods can be used interchangeably. The iridectomy, posterior capsulotomy, and hyaloidotomy may be performed using a vitrectomy tip or using other surgical instruments. In this case series fragments of the circumferential capsule and zonula were removed with a 25-gauge vitrector, and communication was established between the anterior and posterior chamber and vitreous cavity.

During the surgery we pay particular attention to performing vitrectomy in properly wide range in case the vitreous remnants do not disturb the flow through iridotomy and at the end of surgery we avoid leaving the vitreous in the sclerotomies.

At the end of surgery, after the spontaneous deepening of anterior chamber was achieved, the site of the previously performed glaucoma surgery was revised, the anterior chamber was filled using Viscoat, and wounds in the corneal limbus were sealed, sometimes with the application of single Nylon 10/0 sutures.

### 2.4. Postoperative Protocol

Standard ophthalmological examination was performed on days 1 and 7 and 1, 3, 6, and 12 months after surgery. IOP, UDVA, and CDVA (Snellen chart and log⁡MAR) were determined, and the anterior and posterior segments of the eye were examined. Postoperative course was analyzed, including the occurrence of complications and the number of applied antiglaucoma medications.

Complete surgical success was defined for two criteria: IOP ≤ 18 mmHg and IOP ≤ 21 mmHg without antiglaucoma medications, and satisfactory success was defined as IOP at these two levels without and with a maximum of two antiglaucoma medications. Ineffectiveness of the previous filtration procedure was identified when IOP ≥ 21 mmHg was stated or when it was necessary to use antiglaucoma medications [14].

Complications were qualified as “early” if they occurred in the first week after surgery; other complications were qualified as “late” complications. A postoperative IOP ≥ 21 mmHg was considered to be raised. Cystoid macular edema was stated based on deterioration of visual acuity and characteristic biomicroscopic image, and optical coherent tomography (OCT) was performed in order to confirm the diagnosis [15]. Other complications were identified based on clinical picture. A recurrence of malignant glaucoma was considered to be shallowing of the anterior chamber with accompanying progressive IOP increase after malignant glaucoma surgery. In the case of recurrence or suspected obstruction resulting from the formation of fibrin membranes in the area of iridectomy, unblocking of communication between the anterior and posterior segments was performed using Nd:YAG laser with energy 1–5 mJ.

### 2.5. Statistical Analysis

Statistical analysis of the investigated variables was performed with the Shapiro-Wilk and paired Wilcoxon tests. Friedman ANOVA for matched groups and rank means and rank sums were also used for post hoc comparison. The Kaplan-Meier method was used to determine survival curves, and differences between them were tested by the log-rank test. A *P* value of 0.05 or less was considered significant. The calculations were performed with Statistica 10.0 PL.

## 3. Results

### 3.1. Demographic Data

From January 2005 to March 2010 1689 eyes were treated with glaucoma surgery in single clinic (Military Institute of Medicine in Warsaw), 960 (56.8%) with penetrating surgery and 729 (43.2%) with nonpenetrating surgery. 1417 (83.9%) of conducted surgeries were combined with phacoemulsification, while 272 (16.1%) were antiglaucoma surgeries without phacoemulsification. The decision to perform a combined procedure depended on vision loss connected with cataract development, the number of antiglaucoma medications used, and the state of the glaucoma. Subtype of the glaucoma surgery was chosen individually for each patient.

In analyzed material malignant glaucoma occurred in 22 eyes (1.3%). Among patients with penetrating surgery, malignant glaucoma occurred in 2.3% of patients, whereas after nonpenetrating surgery this complication was not found (*P* = 0.00004). The statement was made that penetrating surgery is the risk factor of malignant glaucoma occurrence. The risk of malignant glaucoma after phacotrabeculectomy and phacoiridencleisis was equivalent (*P* = 0.058). When the frequency of malignant glaucoma after trabeculectomy and iridencleisis was compared, the difference was not statistically significant (*P* = 0.416). In the group of patients with malignant glaucoma 40.9% eyes underwent surgical treatment with the method of phacoiridencleisis, 22.7% phacotrabeculectomy, 18.2% iridencleisis, 13.6% trabeculectomy, and 4.5% seton valve implantation before this complication occurred. The phakic eyes with malignant glaucoma and malignant glaucoma cases after cataract surgery were not analyzed for the study purpose.

Twenty eyes of 17 patients (15 women, 2 men) with an average age of 62.9 ± 13.3 (SD) years (Me 64, range: 43–85 years) were recruited into the study. The mean time elapsed from primary glaucoma surgery was about two months but differed markedly equal to 61.4 ± 190.5 (SD) days (Me 2.5; from 1 to 840 days). The mean time of observation was 405 ± 366.1 (SD) days (Me 360; from 7 to 1440 days). Demographic data is presented in Table 1.

### 3.2. Intraocular Pressure Control

**Figure 1:**
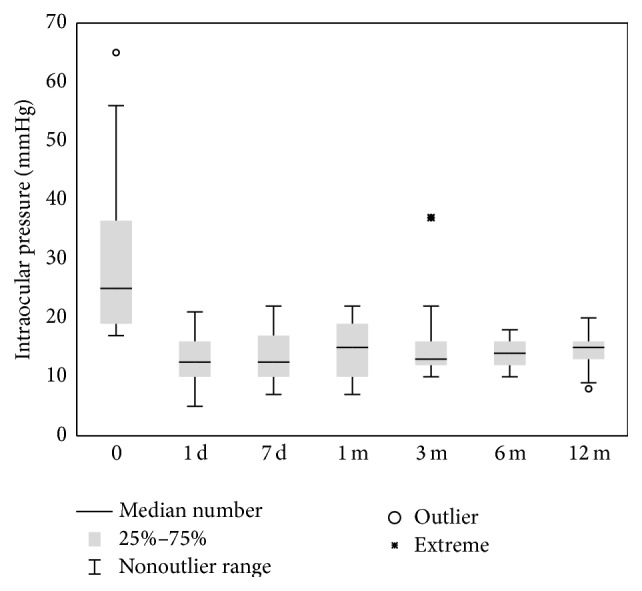
Median number, outlier, and extreme of intraocular pressure at specific time after surgery.

During a 12-month observation period, significant variance of the IOP value was stated (*χ* ^2^_ANOVA_ = 38.73; *P* < 0.001), which resulted from statistically significant IOP reduction on day 1 after surgery (rank means 3.72, rank sum 67.0, and *P* < 0.05) that was maintained until the end of observation (*χ* ^2^_ANOVA_ = 2.51; *P* = 0.77462) (Figure 1, Table 2).

Preoperative mean IOP was 30.4 ± 14.2 (SD) mmHg and decreased during the first day, on average, by 49.3% (range: 11.8–80.8%) to 13.4 ± 4.4 (SD) mmHg (rank means 3.72, rank sum 67.0, and *P* < 0.05). After 12 months of follow-up, mean IOP was 14.6 ± 3.2 (SD) mmHg and was reduced, on average, by 43.0% (range: 5.8–72.3%) relative to initial values (rank means 2.94, rank sum 53.0, and *P* < 0.05) (Table 2).

### 3.3. Medications

Fewer medications were used after surgery than before the procedure. The mean number of antiglaucoma medications applied preoperatively was 3.3 ± 1.1 (SD) (Me 3; range: 1–5), and, at the end of follow-up, this number was significantly reduced to 1.2 ± 1.1 (SD) (Me 1; range: 0–3) (*Z* = 3.059412, *P* = 0.002218) (Table 2).

### 3.4. Surgical Success

**Figure 2:**
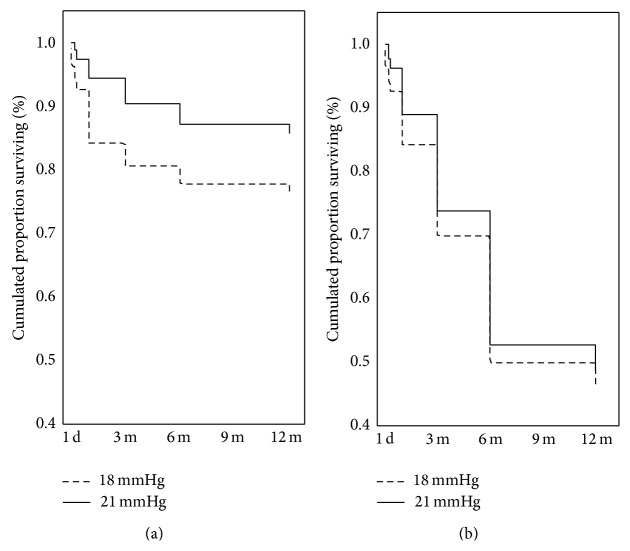
Cumulative surviving proportion (Kaplan-Meier) for success criterion of intraocular pressure less than or equal to 18 and 21 mmHg. (a) Qualified success rate (log-rank test; 2,004523, *P* = 0.04501); (b) complete success rate (log-rank test; 0.7002498, *P* = 0.48377).

The complete success rate after 12 months of follow-up for the criteria 18 and 21 mmHg was, respectively, 46.0% and 49.0%, whereas the qualified success rate was, respectively, 76.2 and 85.7% (Figure 2). It should be noted that a large drop of the percentage of patients fulfilling the complete success criterion is observed after the operation, which is related to the necessity of applying antiglaucoma medications for the purpose of IOP control (Figure 2). In 94.4% of cases, IOP ≤ 18 mmHg was achieved after surgery, and a >30.0% reduction of its value relative to initial values was achieved in 72.2% of cases during the same observation period.

### 3.5. Corrected Distance Visual Acuity

The mean log⁡MAR of CDVA changed from 0.9 ± 0.7 (SD) before surgery to 0.7 ± 0.6 (SD) on day 1 after surgery (rank means 0.50, rank sum 6.50, and *P* > 0.05); the CDVA after 12 months was 0.3 ± 0.5 (SD) (rank means 2.92, rank sum 38.0, and *P* < 0.05) (Table 3).

### 3.6. Complications

The following early complications were observed: raised IOP (5%), inflammatory exudate in the anterior chamber (5%), vitreous hemorrhage (10%), and posterior adhesions (5%). Progressive shallowing of the anterior chamber without increase of IOP was observed in the first week after surgery in 8 cases (40%). Among the late complications, fibrosis was observed in 3 eyes (15.0%), recurrence of malignant glaucoma in 3 eyes (15%), cystoid macular edema in 2 eyes (10%), and retinal detachment in 1 eye (5%).

## 4. Discussion

Despite well-established methods of conservative treatment and laser procedures to treat malignant glaucoma, it is documented that surgical management to release entrapped aqueous fluid remains the most efficacious.

Until Chaundry and coworkers emphasized that establishing patent communication between the anterior chamber and vitreous cavity is the most important part of surgery in cases of malignant glaucoma, pars plana vitrectomy with or without lens extraction was applied to treat malignant glaucoma and was not successful in all cases [5, 6, 12, 16].

The surgical method presented here reflects contemporary views on how to treat malignant glaucoma. Evacuation of vitreous and aqueous humor from the vitreous cavity and establishment of communication with the anterior chamber help stop the vicious mechanism that eventually leads to increased IOP. In postoperative follow-up, it is important to maintain patency of newly created passages by using an Nd:YAG laser, as this helps diminish the number of recurrences. The data presented here clearly demonstrates the significant decrease of IOP on day 1 after surgery and at the end of follow-up. The efficacy of the surgery is further confirmed by the reduction of the number of antiglaucoma medications from about 3 before surgery to about 1 after the operation.

The complete success rate in our whole group of patients for the two criteria of IOP reduction, the IOP ≤ 18 mmHg and IOP ≤ 21 mmHg criteria, was 46% and 45%, respectively, whereas the qualified success rate was 76.2% and 85.7%, respectively. It is noteworthy that there is a great difference between complete and qualified success rates. This is related to the fact that ant-glaucoma drops were frequently necessary to control IOP in our case series.

The most typical and difficult complication of malignant glaucoma surgery is the recurrence of malignant glaucoma. Tsai and coworkers described reoccurrence of symptoms in 33% of pseudophakic eyes treated with PPV and in 75% of phakic eyes after PPV [7]. Lois et al. did not observe recurrence of malignant glaucoma in 5 eyes treated surgically with zonulo-hyaloido-vitrectomy, but the follow-up was relatively short (1–9 months) [12]. Sharma and coworkers also did not note return of malignant glaucoma symptoms in their study, but the procedure of vitrectomy-phacoemulsification-vitrectomy was performed only in 4 eyes [17]. Debrouwere et al. compared percentage of recurrence after conventional vitrectomy (75%) and anterior vitrectomy with iridectomy and zonulectomy (66%), and they did not notice any after total PPV with iridectomy and zonulectomy [18]. In our study, 11 eyes (55%) exhibited isolated shallowing of the anterior chamber after surgical treatment of malignant glaucoma. In all cases, Nd-YAG capsulotomy was performed, and the laser beam was directed through a patent iridotomy/iridectomy or through the pupil. In eight eyes that did not show IOP elevation, deepening of the anterior chamber was achieved. In three eyes with concomitant raised IOP, frank recurrence was regarded; however, only in one case reoperation was needed.

Failure of preceding glaucoma surgery was formerly described in the literature [5, 6, 9, 12]. In our study it was observed in 11 eyes (55%), and this made administration of antiglaucoma drops necessary.

At present, although new methods of treating malignant glaucoma are very promising, it is not possible to totally prevent complications. However, their occurrence can be limited by using new methods and making the decision to treat malignant glaucoma surgically as quickly as possible. Posterior pars plana vitrectomy with concomitant zonulectomy and capsulectomy seems to be efficacious as far as IOP control, post-op visual acuity, and reduction of medications are concerned. In general, it is plausible to perform it without delay before severe complications develop.

The study of malignant glaucoma has its limitations. Its pathomechanism is not fully understood, and the role of communication between the anterior chamber and posterior segment of the eye needs to be elucidated. The onset of malignant glaucoma differs, its frequency is low, and the application of various methods of treatment in groups of patients poses some ethical issues. It seems that further multicenter studies are needed to establish long-term success and optimal instrumentation in a larger group of patients.

## 5. Conclusions

Partial PPV with peripheral lens capsule excision communicating anterior chamber and vitreous cavity allows effective IOP control in 12-month follow-up of 20 operated eyes, although in most cases there was a necessity of additional use of local antiglaucoma treatment in order to obtain the desired level of IOP after operation. In case of recurrences connected with occlusion of created tunnel between anterior chamber and vitreous cavity Nd-Yag laser may be used to restore communication between anterior and posterior segments of the eye. Such a management will usually result in complete resolution of the condition.

## Figures and Tables

**Table 1:** Patients' demographic data.

Demographic data	Mean (SD)	Me	Range
AXL	21.8 ± 0.8	21.9	19.9–23.8
IOL (D)	24.9 ± 2.4	24.5	22.0–32.0
CCT (*µ*m)	539.5 ± 26.9	541.5	492.0–579.0
ACD (mm)	1.8 ± 0.6	2.0	0.5–2.3
c/d	0.8 ± 0.2	0.8	0.3–1.0
CDVA_0_ (log⁡MAR)	0.9 ± 0.7	0.7	0.1–2.3
IOP_0_	30.4 ± 14.2	25.0	17.0–65.0

AXL: axial length; IOL: intraocular lens; CCT: central corneal thickness; ACD: anterior chamber depth; c/d: cup/disc ratio; CDVA_0_: corrected distance visual acuity before surgery; IOP_0_: intraocular pressure before surgery; SD: standard deviation; Me: median number.

**Table 2:** Mean values, median number, standard deviations, ranges of intraocular pressure, and number of medications at specific times after surgery.

Time	Intraocular pressure (mmHg)				Medications (*n*)			
	Mean (SD)	Me	Range	*P* ^∧^	Mean (SD)	Me	Range	*P* ^*∗*^
Pre-op	30.4 ± 14.2	25	17–65		3.3 ± 1.1	3	1–5	
1st day	13.4 ± 4.4	12.5	5–21	<0.05	0.0 ± 0.0	0	—	0.000089
7th day	13.3 ± 4.4	12.5	7–22	<0.05	0.0 ± 0.2	0	0-1	0.000089
1st month	14.6 ± 4.6	15	7–22	<0.05	0.5 ± 1.0	0	0–3	0.000196
3rd month	15.2 ± 6.1	13	10–37	<0.05	0.7 ± 1.1	0	0–4	0.000196
6th month	14.4 ± 2.5	14	10–18	<0.05	0.9 ± 1.0	1	0–3	0.000982
12th month	14.6 ± 3.2	15	8–20	<0.05	1.2 ± 1.1	1	0–3	0.002218

SD: standard deviation; *n*: number of medications; Friedman ANOVA (*χ* ^2^_ANOVA_ = 38.73; *P* < 0.001); post hoc test (rank means, rank sum)^∧^; Wilcoxon signed-rank test^*∗*^; Me: median number.

**Table 3:** Mean values, median number, standard deviations, ranges of corrected distance visual acuity at specific times after surgery.

Corrected distance visual acuity (log⁡MAR)				
Time	Mean (SD)	Me	Range	*P* ^*∗*^
Pre-op	0.9 ± 0.7	0.7	0.1–2.3	
1st day	0.7 ± 0.6	0.5	0–2.3	>0.05
7th day	0.7 ± 0.7	0.3	0–2.3	>0.05
1st month	0.5 ± 0.7	0.3	0–2.3	>0.05
3th month	0.3 ± 0.4	0.2	0–1.9	<0.05
6th month	0.3 ± 0.5	0.1	0–1.9	<0.05
12th month	0.3 ± 0.5	0.2	0–1.9	<0.05

SD-standard deviation; log⁡MAR-*logarithm* of the *minimum angle* of *resolution*; *Friedman* ANOVA (*χ* ^2^_ANOVA_ = 35.19; *P* < 0.001)-post-hoc test (rank means, rank sum)^*∗*^, Me-median number.
